# Evaluation of the AiDx Assist device for automated detection of *Schistosoma* eggs in stool and urine samples in Nigeria

**DOI:** 10.3389/fpara.2025.1440299

**Published:** 2025-03-17

**Authors:** Brice Meulah, Pytsje T. Hoekstra, Samuel Popoola, Satyajith Jujjavarapu, Moses Aderogba, Joseph O. Fadare, John A. Omotayo, David Bell, Cornelis H. Hokke, Lisette van Lieshout, Gleb Vdovine, Jan Carel Diehl, Temitope Agbana, Louise Makau-Barasa, Jacob Solomon

**Affiliations:** ^1^ Leiden University Center for Infectious Diseases, Leiden University Medical Center, Leiden, Netherlands; ^2^ Centre de Recherches Médicales des Lambaréné (CERMEL), Lambaréné, Gabon; ^3^ AiDx Medical BV, Pijnacker, Netherlands; ^4^ The Ending Neglected Diseases (END) Fund, New York, NY, United States; ^5^ College of Medicine, Ekiti State University, Ado Ekiti, Nigeria; ^6^ Consultant, Lake Jackson, TX, United States; ^7^ Mechanical, Maritime and Materials Engineering, Delft University of Technology, Delft, Netherlands; ^8^ Industrial Design Engineering, Delft University of Technology, Delft, Netherlands; ^9^ Neglected Tropical Disease (NTD) Division, Federal Ministry of Health, Abuja, Nigeria

**Keywords:** *Schistosoma haematobium*, *Schistosoma mansoni*, automated digital microscopy, schistosomiasis, diagnosis, Nigeria

## Abstract

**Introduction:**

Schistosomiasis is a public health concern and there is a need for reliable field-compatible diagnostic methods in endemic settings. The AiDx Assist, an artificial intelligence (AI)-based automated microscope, has shown promising results for the detection of *Schistosoma haematobium* eggs in urine. It has been further developed to detect *Schistosoma mansoni* eggs in stool.

**Methods:**

In this study, we evaluated the performance of the AiDx Assist for the detection of *S. mansoni* eggs in stool samples and further validated the performance of the AiDx Assist for the detection of *S. haematobium* eggs in urine samples. Additionally, the potential of the AiDx Assist for the detection of other helminths in stool samples was explored. In total, 405 participants from an area endemic for both *S. mansoni* and *S. haematobium* provided stool and urine samples which were subjected to AiDx Assist (semi- and fully automated), while conventional microscopy was used as the diagnostic reference.

**Results:**

Only samples with complete test results were included in the final analysis, resulting in 375 stool and 398 urine samples, of which 38.4% and 65.3% showed *Schistosoma* eggs by conventional microscopy. The collected images of the stool samples were retrospectively examined for other helminth eggs via manual analysis. For the detection of S. mansoni eggs, the sensitivity of the semi-automated AiDx Assist (86.8%) was significantly higher compared to the fully automated AiDx Assist (56.9%) while the specificity was comparable, with 81.4% and 86.8%, respectively. Retrospectively, eggs of *Ascaris lumbricoides* and *Trichuris trichiura* were visualized. For the examination of urine samples, a comparable sensitivity in the detection of *S. haematobium* eggs was found between the semi-and the fully automated modes of the AiDx Assist, showing 94.6% and 91.9%, respectively. Furthermore, the specificity was comparable, with 90.6%and 91.3% respectively.

**Discussion:**

The AiDx Assist met the World Health Organization Target Product Profile criteria in terms of diagnostic accuracy for the detection of *S. haematobium* eggs in urine samples and performed modestly in the detection of *S. mansoni* eggs in stool samples. With some further improvements, it has the potential to become a valuable diagnostic tool for screening multiple helminth parasites in stool and urine samples.

## 1 Introduction

Schistosomiasis is a neglected tropical disease and a public health concern in endemic settings (Wiegand et al., 2021). Conventional microscopy is the reference method for the diagnosis of schistosomiasis. It involves the detection and quantification of *Schistosoma* eggs in stool or urine samples (WHO, 2002). The need for trained experts to perform this method limits its wide application in field settings. Furthermore, the high demands for microscopy expertise not met by the number of trained microscopists in endemic settings highlights the need for high throughput methods (Chala, 2023; Boonyong et al., 2024). Automating microscopy methods such that the dependency on trained experts is reduced could be a solution.

Several automated microscopes with embedded artificial intelligence (AI) algorithms have been developed for detecting *Schistosoma mansoni* or *Schistosoma haematobium* eggs (Meulah et al., 2022; Oyibo et al., 2022; Sangameswaran, 2022; Ward et al., 2022; Makau-Barasa et al., 2023; Meulah et al., 2023). To the best of our knowledge, none of these microscopes have been validated as a single system for the detection and quantification of both *S. mansoni* and *S. haematobium* eggs in stool and urine respectively, within a field setting.

The AiDx Assist is a low-cost and compact automated microscope with integrated AI. It is relatively easy to use without the need for high-level training compared to conventional microscopy (Onasanya et al., 2023) and has been validated for the diagnosis of *S. haematobium* infection in rural endemic settings in two modes: semi-automated and fully automated modes (Makau-Barasa et al., 2023). In the semi-automated mode, the AI algorithm is disabled and the parasite count is detected and counted by an expert based on a visual examination of images registered by the device. Operations in the fully automated mode, however, include automated parasite detection and counting by the integrated AI algorithm. The design of the AiDx Assist makes it possible for it to be developed and customized for the detection of different parasites in the same or different sample types.

The AiDx Assist has been shown to be a promising diagnostic tool for urogenital schistosomiasis (Makau-Barasa et al., 2023) and could also have great potential for future and timely diagnosis of intestinal schistosomiasis. Since the first evaluation for the detection of *S. haematobium* eggs in Nigeria, the device has been further developed for the detection of *S. mansoni* eggs on Kato–Katz (KK) slides and now requires validation in an endemic setting. In the current study, we carried out a validation of the AiDx Assist, demonstrating its performance in detecting *S. mansoni* and *S. haematobium* eggs in stool and urine samples collected in Nigeria in a setting endemic for both *Schistosoma* species. We also explored the potential of the AiDx Assist to detect other helminth parasites in stool samples.

## 2 Methods

### 2.1 Study design

This cross-sectional study was conducted in local communities of the Federal Capital Territory, Abuja, Nigeria with known endemicity for *S. mansoni* and *S. haematobium* infections. The number of egg-positive samples needed to achieve an assumed sensitivity and specificity of 90% using conventional microscopy as the reference was calculated to be approximately 130 (Jones et al., 2003). A school-based approach was employed for sampling participants across five communities based on the schistosomiasis prevalence data (approximately 40%) obtained from the database of the Neglected Tropical Disease Division of the Federal Ministry of Health. To attain 130 positive samples, the sampling of 325 participants was aimed for. Participants aged 5 years or older were eligible to take part in the study.

### 2.2 Ethics statement

Ethical approval for this study was obtained from the Federal Capital Territory (FCT, Nigeria), Health Research Ethics Committee (HREC). Before collecting samples, written consent was obtained from adults and from the parents or legal guardians of children who wanted to take part, which was confirmed by their signatures. All participants older than 5 years old were included in the study. To safeguard the confidentiality and anonymity of the results, distinct codes were assigned to each of the samples. Following sample collection, mass treatment with praziquantel was administered to all communities according to local guidelines by the NTD unit of the public health department.

### 2.3 Sample processing

**Figure 1:**
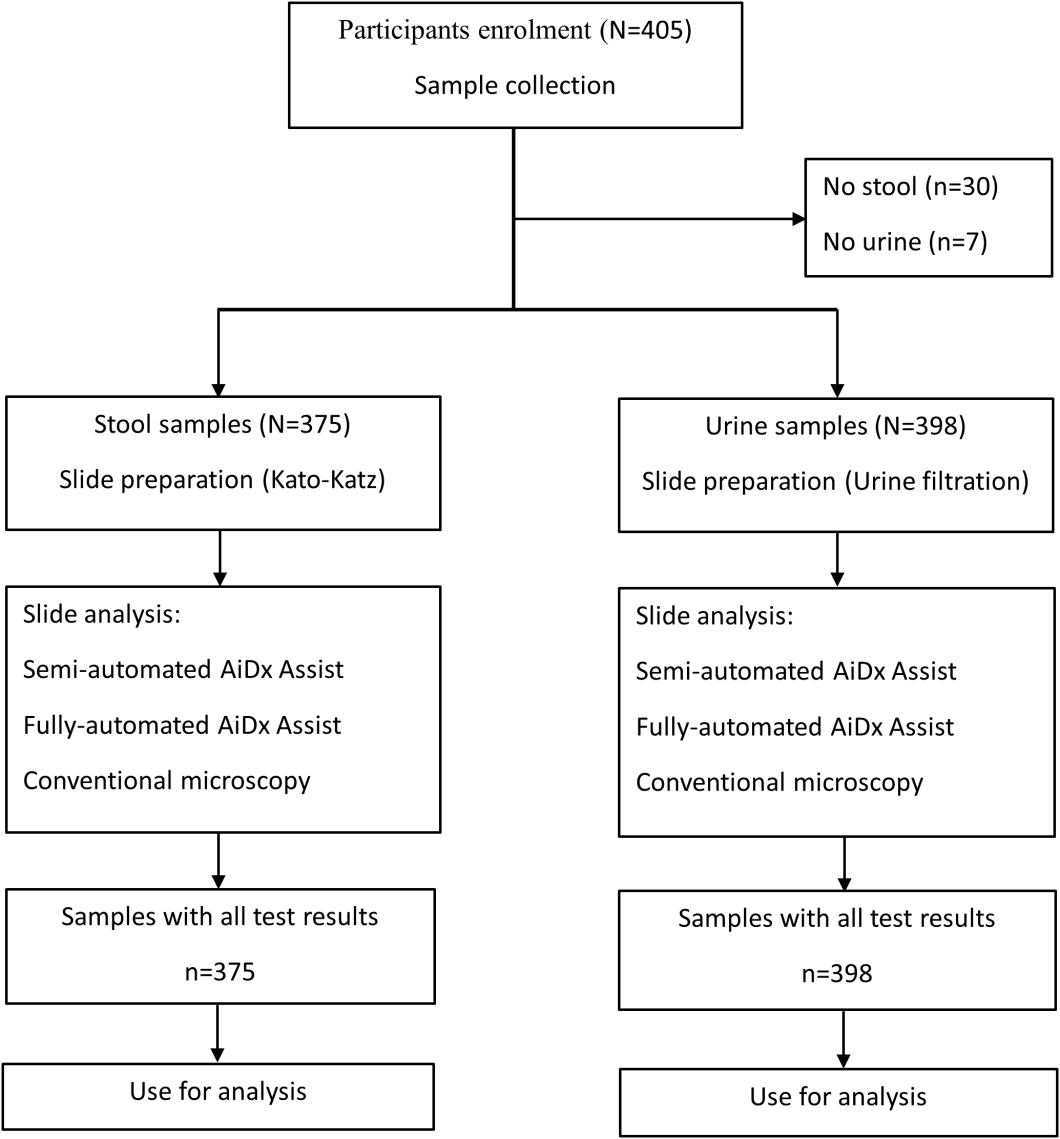
Flowchart of stool and urine sample collection and processing and analyses by the AiDx Assist and conventional microscopy.

A total number of 405 participants were enrolled in this study with an age range of 5 to 14 years. Each participant was given two sterile containers to provide a stool and urine sample at designated collection sites. These samples were then transported in appropriate boxes within 2 hours of collection to the laboratory. From the stool sample, KK slides were prepared (Katz et al., 1972): 41.7mg of sieved stool was transferred to a microscopy slide using a template and then covered with cellophane, which had been dipped in malachite green overnight. Some light pressure was applied to the slide, in order to spread out the smear, and the examination started after 10 minutes. For the urine sample, microscopy slides were prepared by urine filtration (UF). Briefly, 10 ml of homogenized urine was pressed through a 13 mm polycarbonate membrane (pore size 30 µm; Whatmann International Ltd) using a syringe and a filter holder and transferred onto a glass slide. The slides were examined using the semi- and fully automated modes of the AiDx Assist and conventional microscopy (**Figure 1**).

### 2.4 Slide examination by the AiDx Assist and conventional microscopy

**Figure 2:**
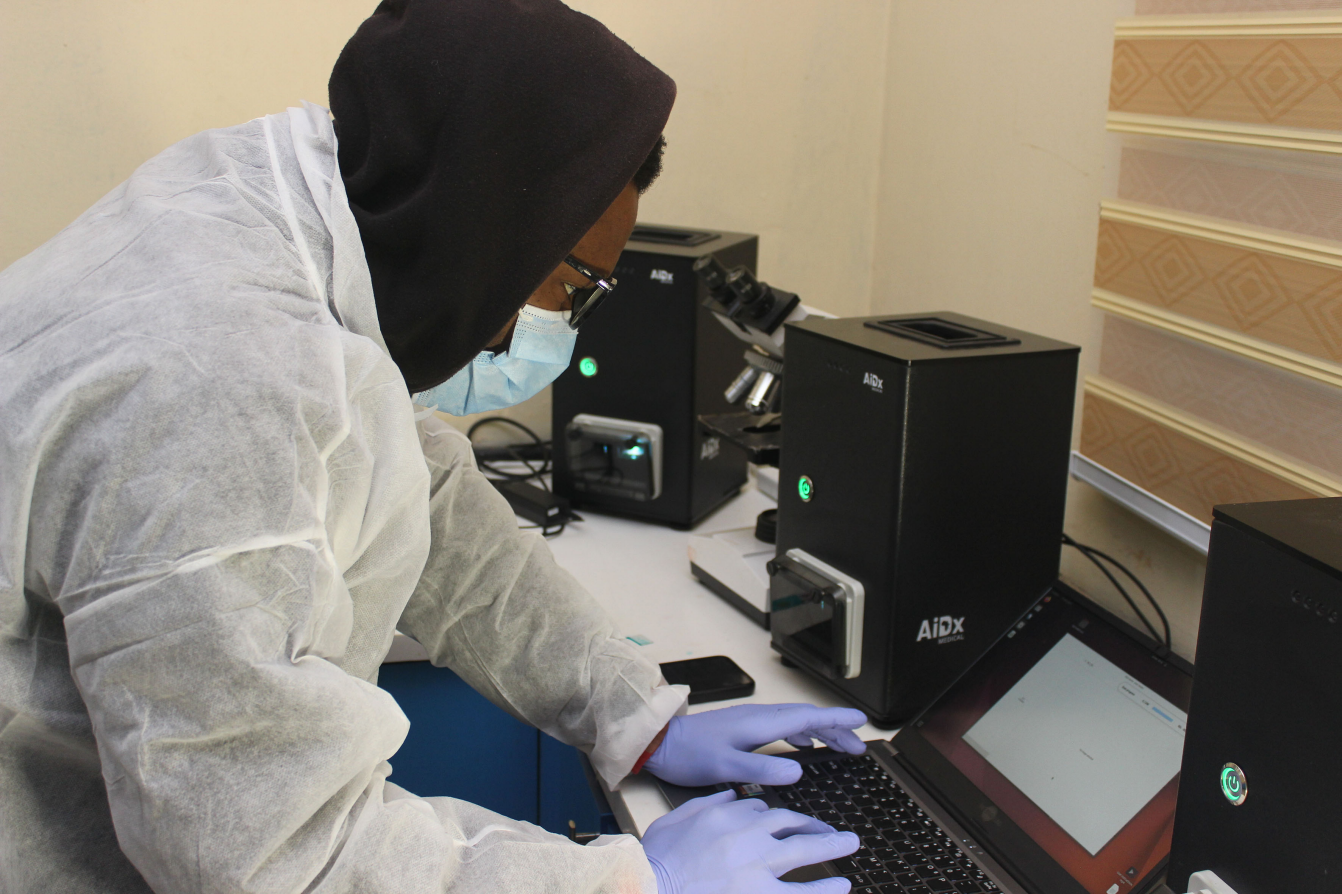
AiDx Assist digital microscope.

Each KK and UF slide was analyzed with the AiDx Assist (**Figure 2**) in the semi-automated and fully automated modes, as previously described (Makau-Barasa et al., 2023). In the semi-automated mode, the images registered by the AiDx Assist were visually examined by an expert for the presence of *Schistosoma* eggs and counted. In the fully automated mode, the artificial intelligence algorithm was enabled to automatically detect and count *Schistosoma* eggs in the images. The output of the AI algorithm was displayed and confirmed by the operator of the device at the end of each sample analysis. The results from the AiDx Assist were exported in an Excel-compatible format. The same slides were subsequently analyzed by conventional microscopy (10/40× objective on a Leica Microsystems DM 300 microscope). Two microscopy readings per slide were performed and recorded independently by two microscopists. The average of the eggs counted in the two readings was considered for each sample. Quality control was performed retrospectively on images captured with the AiDx assist and for the presence of other stool parasites on KK slides on a selected sample with high infection intensity based on conventional microscopy.

### 2.5 Statistical analysis

The number of eggs counted for all methods from the KK and UF slides was expressed in eggs per gram (EPG) of stool and eggs per 10 ml of urine, respectively. Samples were categorized into different infection intensities according to WHO guidelines (WHO, 2002). The percent positive by the AiDx Assist (semi- and fully automated modes) and conventional microscopy was determined. The sensitivity and specificity of the AiDx Assist (semi- and fully automated) were assessed using conventional microscopy as a reference. Cohen’s Kappa (k) statistics were computed to assess the qualitative agreement between methods. Spearman’s correlation (r) was used to assess the pairwise strength of association between eggs counted by the different methods. All statistical analyses were performed using IBM Statistical Package for Social Sciences version 25 (SPSS Inc., Chicago, United States of America) and graphs were generated using GraphPad Prism version 9.0.1 for Windows (GraphPad Software, San Diego, California USA).

## 3 Results

### 3.1 AiDx Assist performance for the detection of *S. mansoni* eggs on Kato-Katz slides

**Figure 3:**
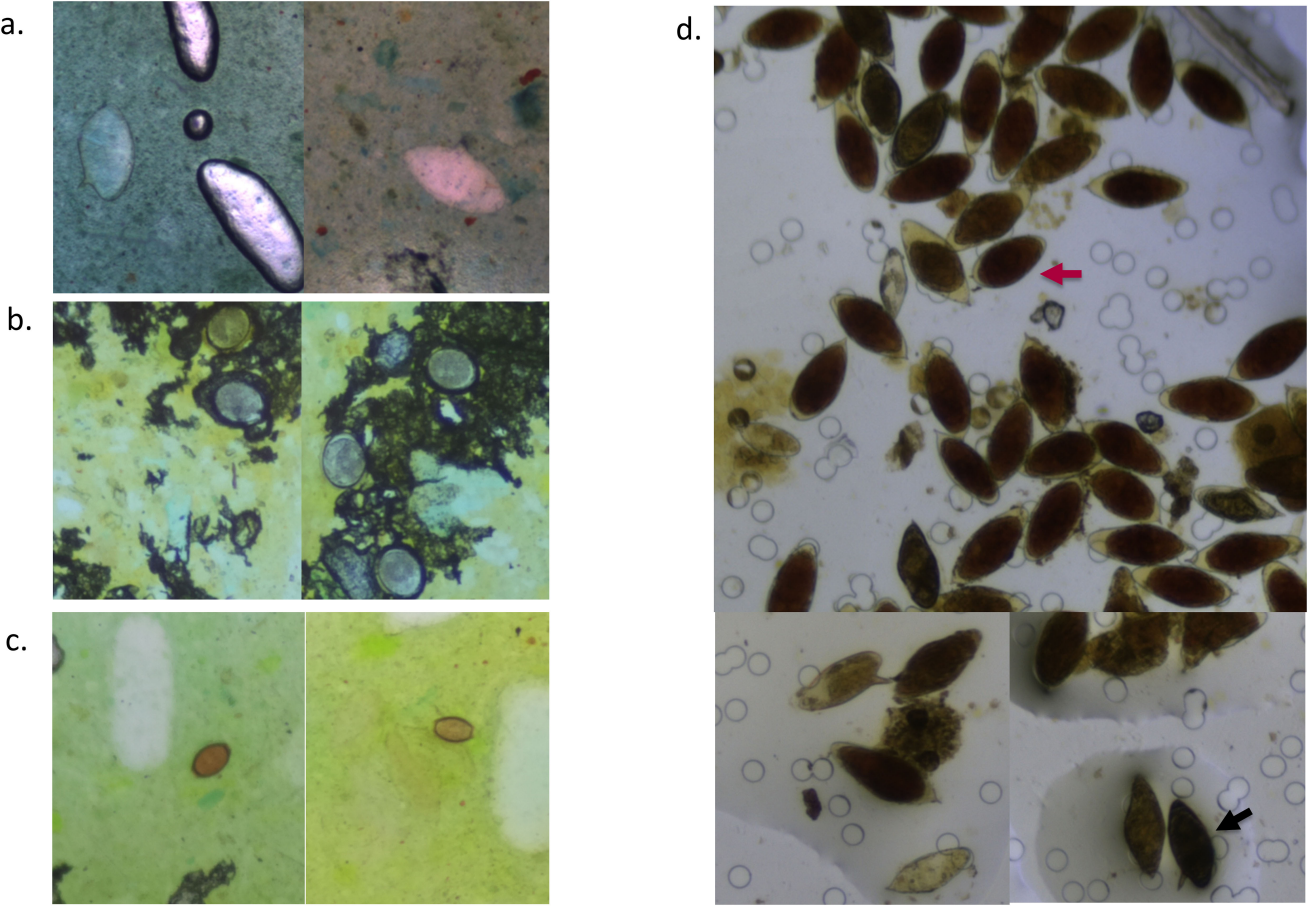
Digital images of Kato–Katz smears **(a–c)** and a urine filtration slide **(d)** captured with the AiDx Assist showing *S. mansoni* eggs **(a)**, *A. lumbricoides* eggs **(b)**, *T. trichiura* eggs **(c)**, and several *S. haematobium* eggs (indicated with a red arrow) and *S. mansoni* eggs (indicated with a black arrow) **(d)**.

A total of 375 stool samples had results for all diagnostic methods and were therefore included in the final analysis (**Figure 1**). **Table 1** shows the proportion of positive samples per method for *S. mansoni* infection. The semi-automated AiDx Assist found the highest proportion of positive samples (44.8%) followed by conventional microscopy (38.4%) and then the fully automated AiDx Assist (25.9%). Different median egg counts were observed for the semi-automated (96 EPG) and fully automated AiDx Assist (48 EPG) and for conventional microscopy (72 EPG). In addition to *S. mansoni*, *Ascaris lumbricoides* and *Trichuris trichiura* eggs were manually detected on digital images captured with the AiDx Assist following retrospective image analysis (**Figures 3a–c**, respectively).

**Table 1:** Characteristic outcomes of the semi-automated and fully-automated AiDx Assist in comparison to conventional microscopy for *Schistosoma* egg detection.

	*S. mansoni* (N=375)			*S. haematobium* (N=398)		
Tests	Semi-automated AiDx-assist	Fully automated AiDx-assist	Conventional microscopy	Semi-automated AiDx-assist	Fully automated AiDx-assist	Conventional microscopy
Positive samples (%)	168 (44.8%)	97 (25.9%)	144 (38.4%)	259 (65.1%)	251 (63.1%)	260 (65.3%)
Low intensity (%)	85 (50.6%)	74 (76.3%)	99 (68.8%)	170 (65.6%)	173 (68.9%)	179 (68.8%)
Moderate intensity (%)	46 (27.4%)	20 (20.6%)	39 (27.0%)	_	_	_
High intensity (%)	37 (22.0%)	3 (3.1%)	6 (4.2%)	89 (34.4%)	78 (31.1%)	81 (31.2%)
Range	24-2304 EPG	24-792 EPG	24- 576 EPG	1-1483 eggs/10ml	1-1169 eggs/10ml	1-181 eggs/10ml
Median	96 EPG	48 EPG	72 EPG	18 eggs/10ml	19 eggs/10ml	16 eggs/10ml

**Figure 4:**
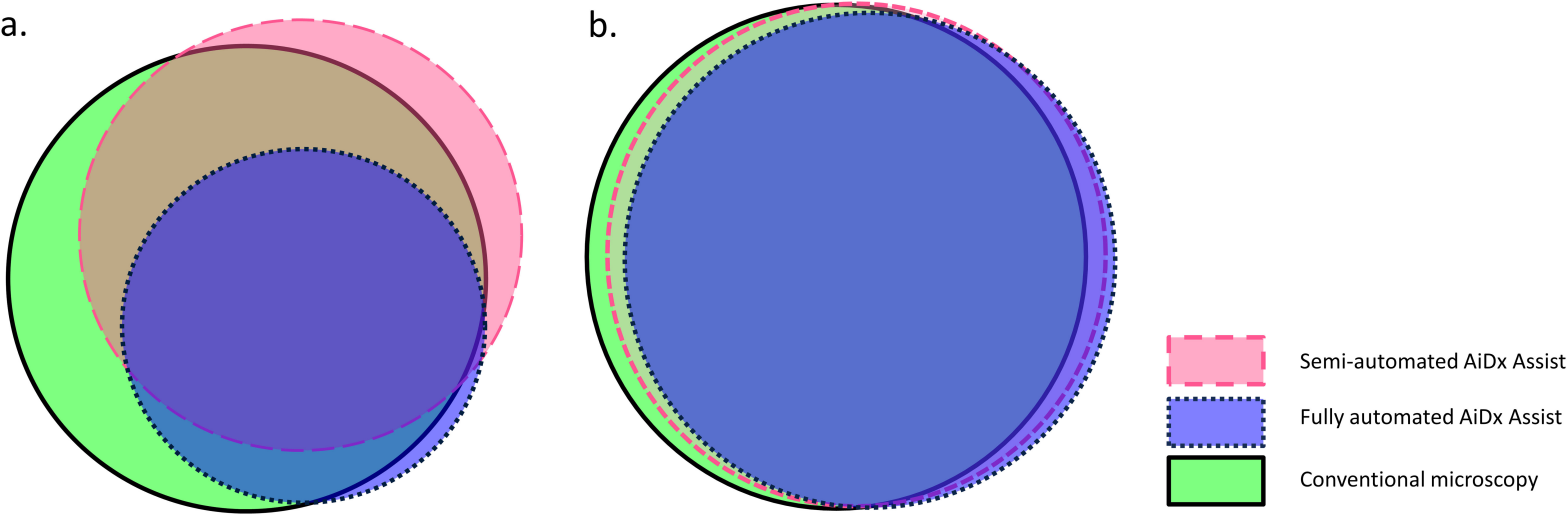
Proportional Venn diagram demonstrating agreement of percent positive using the semi-automated AiDx Assist, fully automated AiDx Assist, and conventional microscopy for the detection of *S. mansoni* and *S. haematobium* eggs in **(a)** stool and **(b)** urine samples, respectively.

Qualitatively, the agreement between the semi-automated AiDx Assist and conventional microscopy was moderate (k=0.538, P<0.05), while the agreement between the fully automated AiDx Assist and conventional microscopy was substantial (k=0.661, P<0.05) for the detection of *S. mansoni* eggs. **Figure 4a** illustrates the diagnostic agreement between conventional microscopy and the semi- and fully automated modes of the AiDx Assist. Based on conventional microscopy, the sensitivity of the semi-automated AiDx Assist (86.8%) for the detection of *S. mansoni* eggs was significantly higher than the fully automated AiDx Assist (56.9%) although their specificities were comparable (81.4% and 86.8%, respectively).

**Figure 5:**
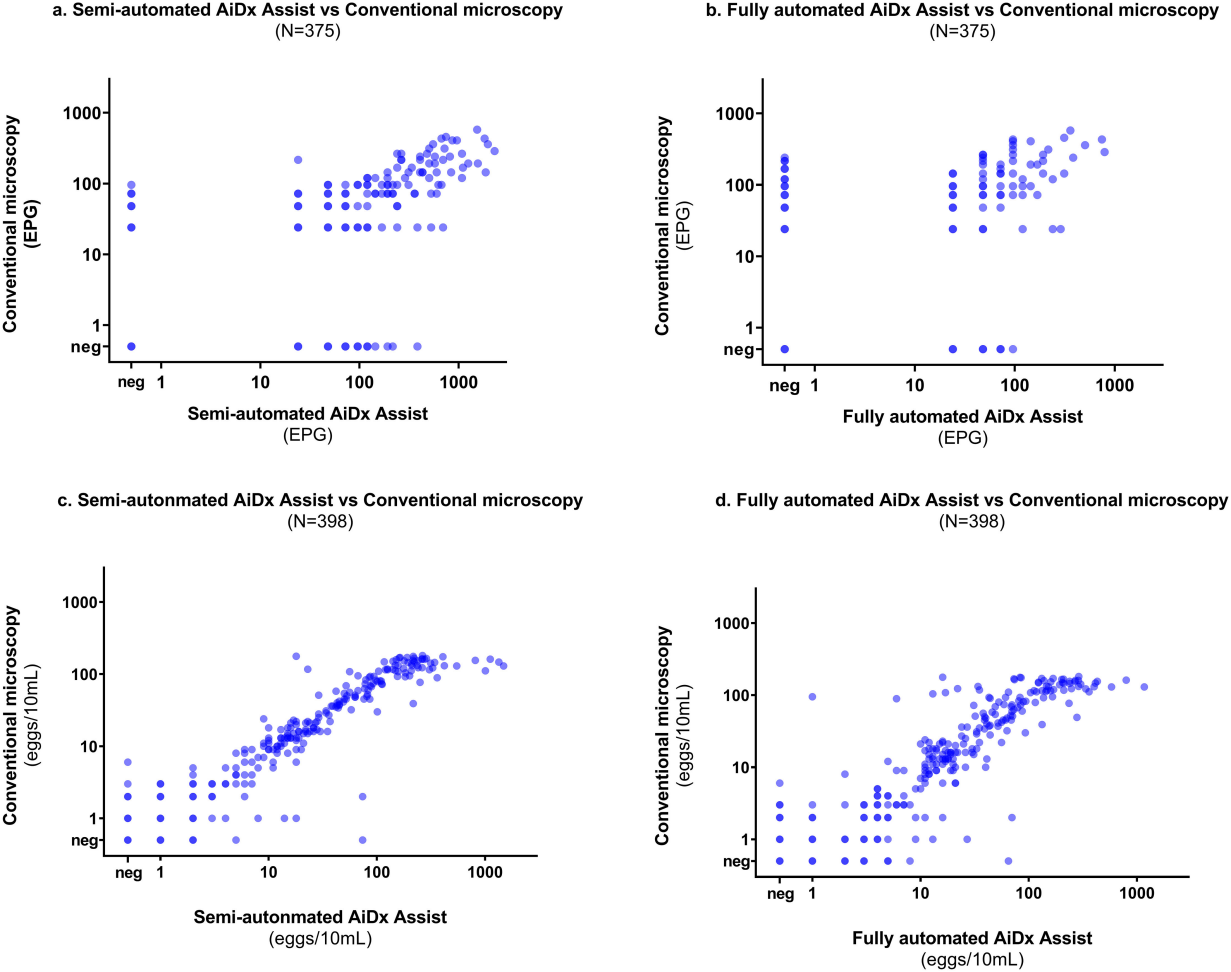
Correlation plot on a log scale between the semi-automated AiDx Assist, fully automated AiDx Assist, and conventional microscopy for **(a, b)** *S. mansoni* egg count **(c, d)** *S. haematobium egg* count.

A moderate correlation was observed between the semi- and fully automated modes of the AiDx Assist and conventional microscopy for the quantification of *S. mansoni* eggs (r = 0.64 P<0.05, r = 0.78, P<0.05 respectively) (**Figures 5a, b**).

### 3.2 AiDx Assist performance for the detection of *S. haematobium* eggs on urine slides

A total of 398 urine samples had results for all three diagnostic methods (**Figure 1**). The proportion of *S. haematobium* infection was comparable among the semi- and fully automated AiDx Assist modes and conventional microscopy (65.1%, 63.1%, and 65.3% respectively) (**Table 1**). The median egg count for *S. haematobium* in all three methods, i.e., the semi-automated and fully automated AiDx Assist modes and conventional microscopy, were comparable (18 eggs/10ml, 19 eggs/10ml, and 16 eggs/10ml, respectively). **Figure 3d** shows digital images of urine slides in which *S. haematobium* and *S. mansoni* eggs are visualized.

An almost perfect agreement was observed between the semi-automated AiDx Assist and conventional microscopy (K=0.820, P<0.05) and between the fully-automated AiDx Assist and conventional microscopy (K=0.851, P<0.05) for the detection of *S. haematobium* eggs (**Figure 3b**). Using conventional microscopy as the reference, the sensitivity and specificity of the semi-automated (94.6% and 90.6%, respectively) and fully automated AiDx Assist modes (91.9% and 91.3%, respectively) for the detection of *S. haematobium* eggs were comparable (**Table 2**).

**Table 2:** Diagnostic performance of the semi-automated and fully-automated AiDx Assist for the detection of *Schistosoma* eggs performed on urine and stool.

	*S. mansoni*		*S. haematobium*	
Index test	Sensitivity % (95% CI)	Specificity % (95% CI)	Sensitivity % (95% CI)	Specificity % (95% CI)
Semi-automated AiDx Assist	86.8 (80.2-91.9)	81.4 (75.8-86.2)	94.6 (91.1-97.0)	90.6 (84.4-94.9)
Fully automated AiDx Assist	56.9 (48.4-65.2)	86.8 (80.2-91.9)	91.9 (87.9-94.9)	91.3 (85.3- 95.4)

Finally, a very strong correlation was observed between egg counts of the semi- and fully-automated AiDx Assist modes and conventional microscopy (r = 0.93 P<0.05, r = 0.95 P<0.05, respectively) (**Figures 5c, d**).

## 4 Discussion

For the first time, we report the performance of the semi- and fully automated AiDx Assist modes as a single device in a field setting for detecting and quantifying *S. mansoni* and *S. haematobium* eggs in stool and urine samples, respectively, using conventional microscopy as the reference. Overall, the performance of the semi-automated and fully automated AiDx Assist modes was found to be modest for the detection of *S. mansoni* eggs and requires optimization to improve the performance. Furthermore, the performance of the semi-automated and fully automated AiDx Assist modes for the detection of *S. haematobium* eggs in urine samples was consistent with Makau-Barasa et al (Makau-Barasa et al., 2023). We also demonstrated that the AiDx Assist has a multi-diagnostic potential as other helminth eggs were visualized in digital images of some of the stool samples.

The significantly higher sensitivity of the semi-automated AiDx Assist (86.8%) over the fully automated AiDx Assist (56.9%) for the detection of *S. mansoni* eggs on KK slides could be due to the fact that stool samples often contain organic matter or artifacts that interfere with the AI detection algorithm, while a trained expert would be able to ignore this and only detect *S. mansoni* eggs. This is a phenomenon also observed by Dacal et al (Dacal et al., 2021). Another reason could be the difference in stool properties such as color, texture, and consistency and variation in stain preparation leading to variable KK smears (as observed in **Figures 3a–c**). The AiDx Assist AI algorithm for the detection of *S. mansoni* eggs in stool samples requires optimization, and such factors need to be taken into consideration. Thus, a high-quality and diverse data set covering as many different KK smear variations as possible is required to further train the AI algorithm. Furthermore, providing a ready-to-use stain with the AiDx Assist, accompanied by a certificate of analysis and expiration date, could help reduce variability in Kato–Katz smears. Moreover, validation of the AiDx Assist using alternative stool preparation methods, e.g., the floatation preparation method that results in relatively clean slides, could be a solution to the challenges related to organic matter or artifacts. However, such methods are not field-compatible (Meurs et al., 2017; Khurana et al., 2021).

Additional analysis revealed that the majority of the stool samples (68.8%) had low infection intensity (1-99 EPG, **Table 1**) based on conventional microscopy, of which more than half were missed by the AI algorithm. Therefore, further optimizing the AI algorithm to accurately detect light infections would significantly improve the overall performance of the fully automated AiDx Assist. The comparable specificity of the semi-automated AiDx Assist (81.4%) to the fully automated AiDx Assist (86.8%) is due to the additional validation step by the operator during the operation of the fully automated AiDx Assist as the positive cases detected by the AI are further checked and ruled out if they are a false positive.

The sensitivity and specificity of the semi-automated AiDx Assist and fully automated AiDx Assist for the detection *S. haematobium* eggs based on conventional microscopy were comparable (**Table 2**) and were consistent with previously reported findings (Makau-Barasa et al., 2023). Despite differences in the positive rates and similarities in the infection intensity observed between studies, the consistent outcome provides more evidence of the potential reliability and robustness of the AiDx Assist for the detection of *S. haematobium* infection in urine. This also provides more evidence that the AiDx Assist meets the required WHO diagnostic Target Product Profile (TPP) (WHO, 2021) for sensitivity and specificity for *S. haematobium.*

Although a strong to moderate correlation was observed between the semi-automated AiDx Assist, the fully automated AiDx Assist, and microscopy for *S. mansoni* and *S. haematobium* egg quantification, at high infection intensities, conventional microscopy tended to underestimate egg count. For example, the upper limit of the *S. haematobium* egg count range for the semi-automated and fully-automated AiDx Assist modes was significantly higher than for conventional microscopy. This observation is contrary to that of Meulah et al. (2022) where, for ≥100 eggs/10ml of urine, the AI algorithm integrated into the Schistoscope underestimated egg counts due to egg overlap. This contradictory observation could be partly due to the differences in AI architecture used and the level of experience of the microscopists between studies. Images of samples with high egg counts (≥100 eggs/10ml for *S. haematobium* and ≥1000 EPG for *S. mansoni*) based on the semi-automated AiDx Asist were re-analyzed manually by an independent experienced microscopist, which confirmed that conventional microscopy underestimated egg counts for samples with high infection intensity. This could be due to the microscopist having less experience in estimating egg count for samples with high infection intensity, highlighting the importance of AI-based digital microscopy in addressing potential gaps between highly and less-experienced microscopists in field laboratory settings. Moreover, a better correlation scatter between the semi- and fully automated AiDx Assist modes was observed across all infection intensities for both *S. mansoni* and *S. haematobium* egg quantification (**Supplementary Figure 1**).

By retrospectively analyzing digital images of KK slides prepared from various samples, it was possible to visualize other stool helminth parasites. The design of the AiDx Assist optical system theoretically enables the visualization of parasite features within a size range of approximately 15-400μm. This implies that parasites and/or their eggs within this size range can be manually detected, as also demonstrated by other studies (Yang et al., 2019; Dacal et al., 2021; Ward et al., 2022; Boonyong et al., 2024; Lundin et al., 2024). However, the current AI-powered prototype of the AiDx Assist has been specifically developed to detect eggs of *S. mansoni* and *S. haematobium*. While visualizing other helminth eggs on digital images captured with the AiDx Assist demonstrates its potential for detecting additional parasites, further development and optimization are necessary for these parasites. This process would involve generating datasets for different stool helminth parasites to train an AI algorithm to recognize these eggs, followed by validation. The digital images generated in this study could serve this purpose. Furthermore, the different variations in the digital image dataset collected in this study could be used to optimize the diagnostic performance of the fully automated AiDx Assist for detecting *S. mansoni* eggs and to develop the device for other helminth parasites in stool samples.

The limitation of this study is the fact that during conventional microscopy, the technicians were only asked to mark the number of *Schistosoma* eggs. Consequently, when exploring the AiDx Assist’s capability to identify other stool helminths through digital images, no comparison could be made to conventional microscopy. This missed opportunity could have provided further evidence regarding the device’s potential to detect other stool parasites.

In conclusion, the overall diagnostic performance of the semi- and fully automated AiDx Assist for the detection of *S. mansoni* infection was found to be modest and requires improvement to meet the WHO TPP in terms of diagnostic accuracy. The consistent performance in *S. haematobium* detection and the additional observation of *A. lumbricoides* and *T. trichiura* revealed its potential for screening multiple diseases in endemic settings.

## Data Availability

The raw data supporting the conclusions of this article will be made available by the authors, without undue reservation.

## Supplementary material

Supplementary Figure 1 Correlation plot on a log scale between the semi-automated AiDx Assist and the fully-automated AiDx Assist for **(a)** *S. mansoni* egg count and **(b)** *S. haematobium egg* count.
