# Supplementary figures and images for: Evaluation of the AiDx Assist device for automated detection of Schistosoma eggs in stool and urine samples in Nigeria
Source: Front Parasitol. 2025 Mar 17;4:1440299. doi: 10.3389/fpara.2025.1440299 (PMC11955702; doi:10.3389/fpara.2025.1440299)

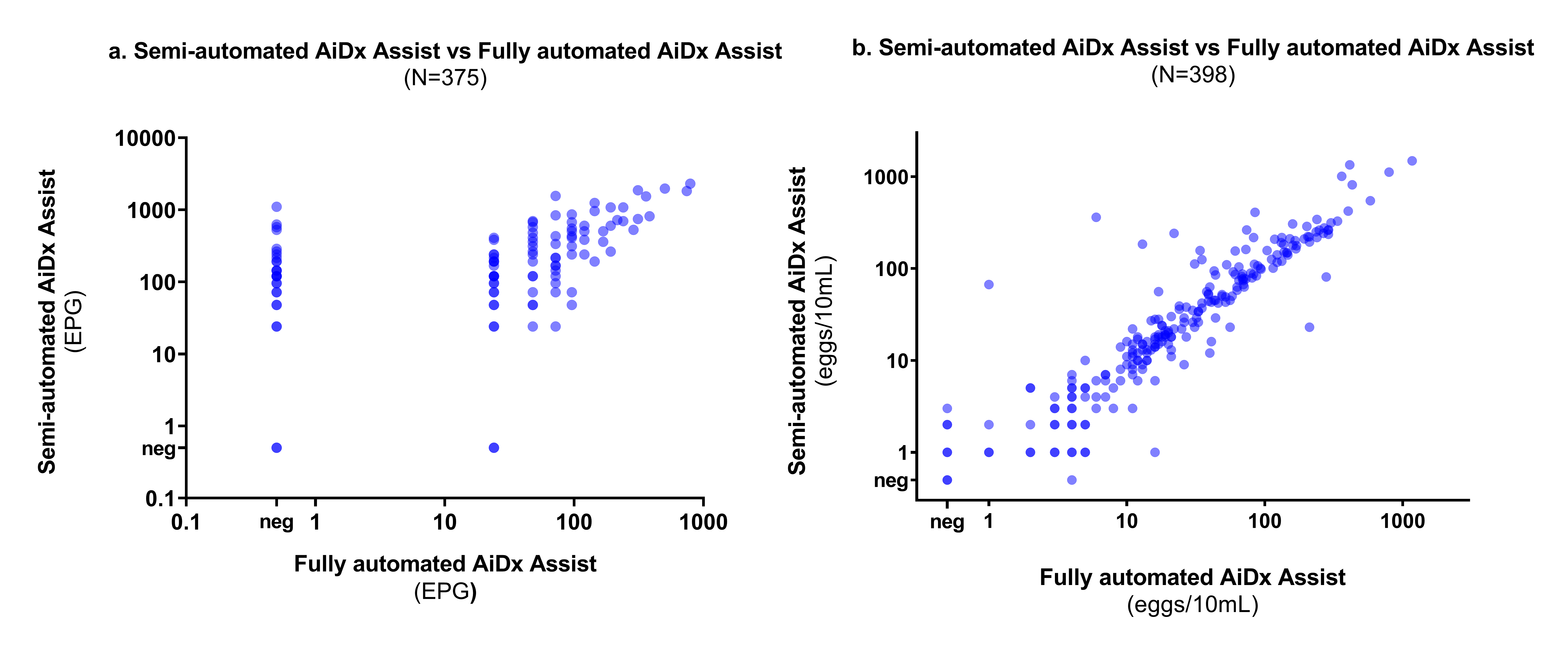

Supplement: Supplementary Figure 1 — Correlation plot on a log scale between the semi-automated AiDx Assist and the fully-automated AiDx Assist for (a) S. mansoni egg count and (b) S. haematobium egg count. [file Image1.tif]
